# Gender-specific impact of personal health parameters on individual brain aging in cognitively unimpaired elderly subjects

**DOI:** 10.3389/fnagi.2014.00094

**Published:** 2014-05-23

**Authors:** Katja Franke, Michael Ristow, Christian Gaser, The Alzheimer's Disease Neuroimaging Initiative

**Affiliations:** ^1^Structural Brain Mapping Group, Department of Psychiatry, Jena University HospitalJena, Germany; ^2^Department of Neurology, Jena University HospitalJena, Germany; ^3^Department of Human Nutrition, Friedrich Schiller-University JenaJena, Germany; ^4^Energy Metabolism Laboratory, ETH Zurich (Swiss Federal Institute of Technology)Schwerzenbach, Zürich, Switzerland

**Keywords:** aging, Alzheimer's disease (AD), *BrainAGE*, lifestyle, magnetic resonance imaging (MRI), voxel-based morphometry (VBM)

## Abstract

Aging alters brain structure and function. Personal health markers and modifiable lifestyle factors are related to individual brain aging as well as to the risk of developing Alzheimer's disease (AD). This study used a novel magnetic resonance imaging (MRI)-based biomarker to assess the effects of 17 health markers on individual brain aging in cognitively unimpaired elderly subjects. By employing kernel regression methods, the expression of normal brain-aging patterns forms the basis to estimate the brain age of a given new subject. If the estimated age is higher than the chronological age, a positive *brain age gap estimation (BrainAGE)* score indicates accelerated atrophy and is considered a risk factor for developing AD. Within this cross-sectional, multi-center study 228 cognitively unimpaired elderly subjects (118 males) completed an MRI at 1.5Tesla, physiological and blood parameter assessments. The multivariate regression model combining all measured parameters was capable of explaining 39% of *BrainAGE* variance in males (*p* < 0.001) and 32% in females (*p* < 0.01). Furthermore, markers of the metabolic syndrome as well as markers of liver and kidney functions were profoundly related to *BrainAGE* scores in males (*p* < 0.05). In females, markers of liver and kidney functions as well as supply of vitamin B_12_ were significantly related to *BrainAGE* (*p* < 0.05). In conclusion, in cognitively unimpaired elderly subjects several clinical markers of poor health were associated with subtle structural changes in the brain that reflect accelerated aging, whereas protective effects on brain aging were observed for markers of good health. Additionally, the relations between individual brain aging and miscellaneous health markers show gender-specific patterns. The *BrainAGE* approach may thus serve as a clinically relevant biomarker for the detection of subtly abnormal patterns of brain aging probably preceding cognitive decline and development of AD.

## Introduction

The global prevalence of dementia is projected to rise sharply over the next decades. By 2050, 1 in 85 persons worldwide will be affected by Alzheimer's disease (AD), the most common form of dementia (Brookmeyer et al., 2007). Manifold pathological changes accumulate over many years or decades before cognitive decline occurs gradually, with dementia representing the final stage of the pathological cascade (Frisoni et al., 2010; Jack et al., 2010). These pathological changes include precocious and/or accelerated brain aging (Fotenos et al., 2008; Driscoll et al., 2009; Sluimer et al., 2009; Wang et al., 2009; Spulber et al., 2010; Clark et al., 2012). Recently, atrophic regions detected in AD patients were found to largely overlap with those regions showing a normal age-related decline in healthy control subjects (Dukart et al., 2011). Hence, early identification of neuroanatomical changes deviating from the normal age-related atrophy pattern has the potential to improve clinical outcomes in the disease course through early treatment or prophylaxis (Ashburner et al., 2003).

Though “healthy” brain aging has been found to follow highly coordinated and sequenced patterns of brain tissue loss and cerebrospinal fluid (CSF) expansion (Pfefferbaum et al., 1994; Good et al., 2001; Resnick et al., 2003; Raz and Rodrigue, 2006; Terribilli et al., 2011), multiple factors affect and modify those individual trajectories. Several markers of poor health and/or inappropriate lifestyle (including obesity, high cholesterol, nicotine and alcohol abuse, hypertension, diabetes, as well as elevated serum total homocysteine (tHcy) and lower levels of vitamin B_12_) have been related to the risk of accelerated brain atrophy, cognitive decline, and even dementia (Clarke et al., 1998, 2007; Ellinson et al., 2004; Clarke, 2006; Steele et al., 2007; Solfrizzi et al., 2008; Chen et al., 2009; Fitzpatrick et al., 2009; Debette et al., 2010; Oulhaj et al., 2010; Zylberstein et al., 2011). Furthermore, the combination of risk factors was found to further boost the risk (Luchsinger et al., 2005). Particularly, components of the metabolic syndrome, i.e., a higher body mass index (BMI), elevated cholesterol and fasting glucose levels, and a higher diastolic blood pressure (DBP), are associated with a greater rate of brain atrophy (Enzinger et al., 2005) as well as an increased risk of dementia (Middleton and Yaffe, 2009).

In contrast, a healthy and well-balanced lifestyle (including physical activity, normal body weight, smoking cessation, Mediterranean diet, and moderate alcohol intake) was shown to lower the risk of cognitive decline and dementia (Peters et al., 2008; Solfrizzi et al., 2008; Luchsinger and Gustafson, 2009; Scarmeas et al., 2009; Xu et al., 2009; Erickson et al., 2010; Feart et al., 2010; Frisardi et al., 2010; Gu et al., 2010; Nepal et al., 2010). Maintaining cardiovascular health in midlife was recently suggested to be the most promising strategy for preventing cognitive impairment and dementia in late life (Hughes and Ganguli, 2009).

Based on the widespread but well-ordered brain tissue loss that occurs with healthy aging into senescence (Good et al., 2001), we previously proposed a modeling approach to identify abnormal aging-related brain atrophy that may precede the onset of cognitive decline and clinical symptoms. We introduced a novel *BrainAGE* approach (Franke et al., 2010, 2012b) based on a database of single time-point structural magnetic resonance imaging (MRI) data that aggregates the complex, multidimensional aging patterns across the whole brain to one single value, i.e., the estimated brain age. The difference between estimated and true chronological age will reveal the individual *brain age gap estimation* (*BrainAGE*) score. Consequently, the *BrainAGE* score directly quantifies subtle deviations in “normal” age-related brain atrophy by analyzing only one standard MRI per subject, with positive *BrainAGE* scores indicating accelerated structural brain aging and negative *BrainAGE* scores indicating decelerated structural brain aging. Recent work has demonstrated that increased *BrainAGE* scores in subjects with mild cognitive impairment (MCI) related to an increased risk of converting to AD, with each additional year in the baseline *BrainAGE* score being associated with a 10% greater risk of converting to AD within the next three years (Gaser et al., 2013). Furthermore, we observed profound relationships between *BrainAGE*, disease severity, prospective worsening of cognitive functions (Franke et al., 2012a), conversion to AD (Gaser et al., 2013), as well as diabetes mellitus type 2 (Franke et al., 2013).

In this study, we implemented the *BrainAGE* method to explore and quantify the effects of several physiological and clinical chemistry markers of personal health on individual *BrainAGE* scores in a subsample of cognitively unimpaired older adults from the Alzheimer's Disease Neuroimaging Initiative (ADNI) database. Since men and women were found to differ in basic aspects of their normal function, their experience of the same illness (Pinn, 2003), probable risk factors on individual brain aging were separately examined in men and women. We expect gender-specific patterns in the relations between *BrainAGE* scores and health markers. Additionally, we expect the combination of the most significant risk factors to be associated with an even greater effect on individual *BrainAGE* scores than each factor independently.

## Methods

### Subjects

To train the age estimation framework, we used T_1_-weighted MRI data of 561 healthy subjects (250 male) from the publicly accessible IXI cohort (http://www.brain-development.org; data downloaded in September 2011) aged 20–86 years [mean (*SD*) = 48.6 (16.5) years], which were collected on three different scanners (Philips 1.5T, General Electric 1.5T, Philips 3.0T). For more sample details see Franke et al. (2010).

The current *BrainAGE* analyses were conducted using data obtained from the ADNI database (www.loni.ucla.edu/ADNI). ADNI utilizes the following diagnostic criteria to classify the subjects into (1) Normal subjects (NO): “Mini-Mental State Examination” (MMSE; Cockrell and Folstein, 1988; test range 0–30) scores between 24 and 30 (inclusive), a “Clinical Dementia Rating” (CDR; Morris, 1993; test range 0–3) score of 0, non-depressed, non-MCI, and non-demented; (2) MCI subjects: MMSE scores between 24 and 30 (inclusive), a memory complaint, objective memory loss measured by education adjusted scores on Wechsler Memory Scale Logical Memory II, a CDR score of 0.5, absence of significant levels of impairment in other cognitive domains, essentially preserved activities of daily living, and an absence of dementia; (3) mild AD: MMSE scores between 20 and 26 (inclusive), CDR scores of 0.5 or 1.0, and meeting NINCDS/ADRDA criteria for probable AD. Detailed description of the ADNI inclusion and exclusion criteria is available at http://www.adni-info.org/Scientists/Pdfs/adniproceduresmanual12.pdf.

Adopting the diagnostic classification from ADNI, we included all subjects who (i) were diagnosed as NO (i.e., cognitively unimpaired) at their baseline visit; for whom (ii) MRI data (1.5T), and (iii) a battery of physiological and clinical chemistry parameters at baseline were available [i.e., albumin, alanin-aminotransferase (ALT), aspartat-aminotransferase (AST), systolic (SBP) and diastolic blood pressure (DBP), BMI, γ-glutamyltransferase (GGT), glucose, mean erythrocyte cell volume (MCV), thyroid stimulating hormone (TSH), triglycerides, total bilirubin, creatinine, tHcy, uric acid, cholesterol, vitamin B_12_; Table 1]. Exact procedures of collection and processing of the physiological, clinical chemistry and MRI data can be found in the “ADNI Procedures Manual” (http://www.adni-info.org/Scientists/Pdfs/adniproceduresmanual12.pdf).

**Table 1:** **Baseline characteristics of all measured physiological and clinical chemistry parameters in male and female test samples**.

	**MALE**			**FEMALE**			***p*-value (male vs. female)**
	***n***	**Mean (*SD*)**	***p*-value (test for normality)**	***n***	**Mean (*SD*)**	***p*-value (test for normality)**	
Albumin (g/dl)	115	4.20 (0.31)	0.012	107	4.16 (0.31)	n.s.	n.s.
ALT (U/l)	115	22.52 (8.62)	0.001	107	18.46 (5.90)	0.001	0.0001
AST (U/l)	112	24.10 (5.75)	0.001	107	23.31 (5.40)	0.001	n.s.
Direct Bilirubin (mg/dl)	112	0.16 (0.07)	0.001	107	0.12 (0.05)	0.001	0.0001
Total Bilirubin (mg/dl)	115	0.61 (0.30)	0.001	107	0.46 (0.20)	0.001	0.0001
SBP (mmHg)	118	134.9 (17.21)	0.018	110	134.1 (16.69)	n.s.	n.s.
DBP (mmHg)	118	75.14 (9.66)	n.s.	110	74.26 (10.90)	n.s.	n.s.
BMI (kg/m^2^)	117	26.76 (3.75)	n.s.	110	26.55 (4.99)	0.001	n.s.
Cholesterol (mg/dl)	116	178.0 (34.21)	0.002	108	209.8 (39.45)	n.s.	0.0001
Creatinine (mg/dl)	116	1.07 (0.23)	0.001	108	0.83 (0.18)	0.001	0.0001
GGT (U/l)	116	26.11 (16.80)	0.001	108	22.53 (15.34)	0.001	0.002
Glucose (mg/dl)	115	104.2 (25.77)	0.001	107	99.9 (15.63)	0.001	n.s.
MCV (fL)	114	90.42 (4.94)	0.03	108	89.26 (5.44)	n.s.	n.s.
TSH (μIU/mL)	115	1.92 (1.20)	0.001	109	2.01 (1.44)	0.001	n.s.
tHcy (μmol/l)	118	10.56 (2.98)	0.001	108	9.29 (2.45)	0.002	0.001
Triglycerides (mg/dl)	116	152.3 (91.9)	0.001	108	133.9 (76.5)	0.001	0.05
Uric Acid (mg/dl)	116	6.03 (1.30)	n.s.	108	5.02 (1.39)	0.04	0.0001
Vitamin B_12_ (ng/l)	113	477.3 (317.1)	0.001	108	600.6 (414.0)	0.001	0.002

*ALT, alanin-aminotransferase; AST, aspartat-aminotransferase; BMI, body mass index; DBP, diastolic blood pressure; GGT, γ-glutamyltransferase; MCV, mean erythrocyte cell volume; SBP, systolic blood pressure; TSH, thyroid stimulating hormone; tHcy, total homocysteine; n.s., not significant*.

Thus, the male test sample consisted of 118 cognitively unimpaired men, aged 60–88 years, with a mean age of 75.8 ± 5.3 years. Mean MMSE at baseline was 29.0 ± 1.1. The female test sample contained 110 cognitively unimpaired women, aged 62–90 years, with a mean age of 76.1 ± 4.8 years. Mean MMSE at baseline was 29.2 ± 0.9. More cognitive test scores for baseline and available follow-up assessments are presented in Table 2. Additionally, a list of subjects included in this study is provided in Supplement 1.

**Table 2:**
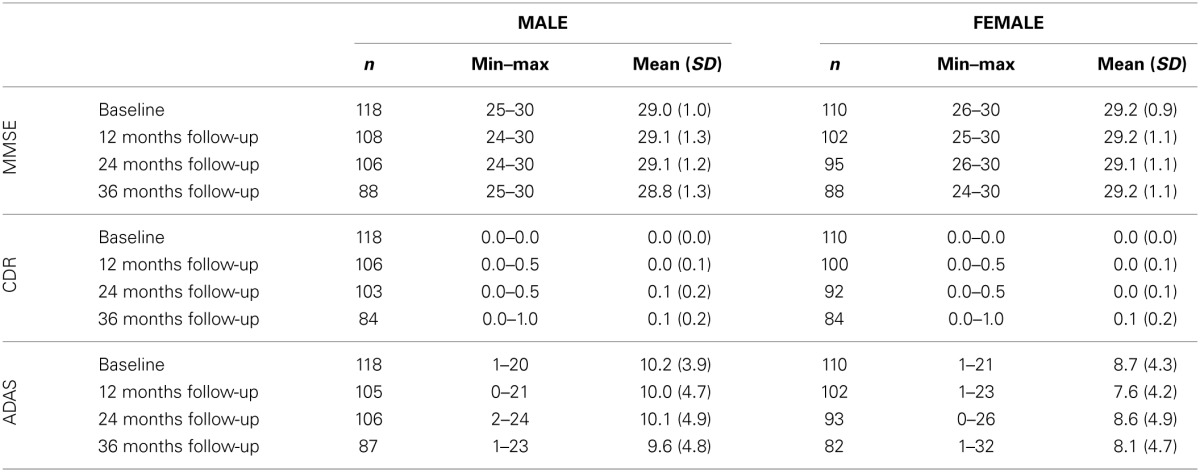
**Cognitive characteristics in male and female test samples**.

*ADAS, Alzheimer's Disease Assessment Scale; CDR, Clinical Dementia Rating; MMSE, Mini-Mental State Examination*.

### Preprocessing of MRI data and data reduction

As described in Franke et al. (2010), preprocessing of the T1-weighted images was done using the SPM8 package (http://www.fil.ion.ucl.ac.uk/spm/) and the VBM8 toolbox (http://dbm.neuro.uni-jena.de), running under Matlab. All T1-weighted images were corrected for bias-field inhomogeneities, then spatially normalized and segmented into gray matter (GM), white matter (WM), and CSF within the same generative model (Ashburner and Friston, 2005). As recently described (Gaser, 2009), the segmentation procedure was further extended by accounting for partial volume effects (Tohka et al., 2004), applying adaptive maximum a posteriori estimations (Rajapakse et al., 1997), and using a hidden Markov Random Field model (Cuadra et al., 2005). After segmentation, only GM images were used. The images were processed with affine registration and smoothed with 8-mm full-width-at-half-maximum (FWHM) smoothing kernels. Spatial resolution was set to 8 mm. Data reduction was performed by applying principal component analysis (PCA), utilizing the “Matlab Toolbox for Dimensionality Reduction” (http://ict.ewi.tudelft.nl/~lvandermaaten/Home.html).

### Age estimation framework

The *BrainAGE* framework utilizes a machine-learning pattern recognition method, namely relevance vector regression (RVR; Tipping, 2001). It was recently developed to model healthy brain aging and subsequently estimate individual brain ages based on T1-weighted images (Franke et al., 2010). As suggested by Franke et al. (2010), the kernel was chosen to be a polynomial of degree 1, since age estimation accuracy was shown to not improve when choosing non-linear kernels. Thus, parameter optimization during the training procedure was not necessary.

**Figure 1:**
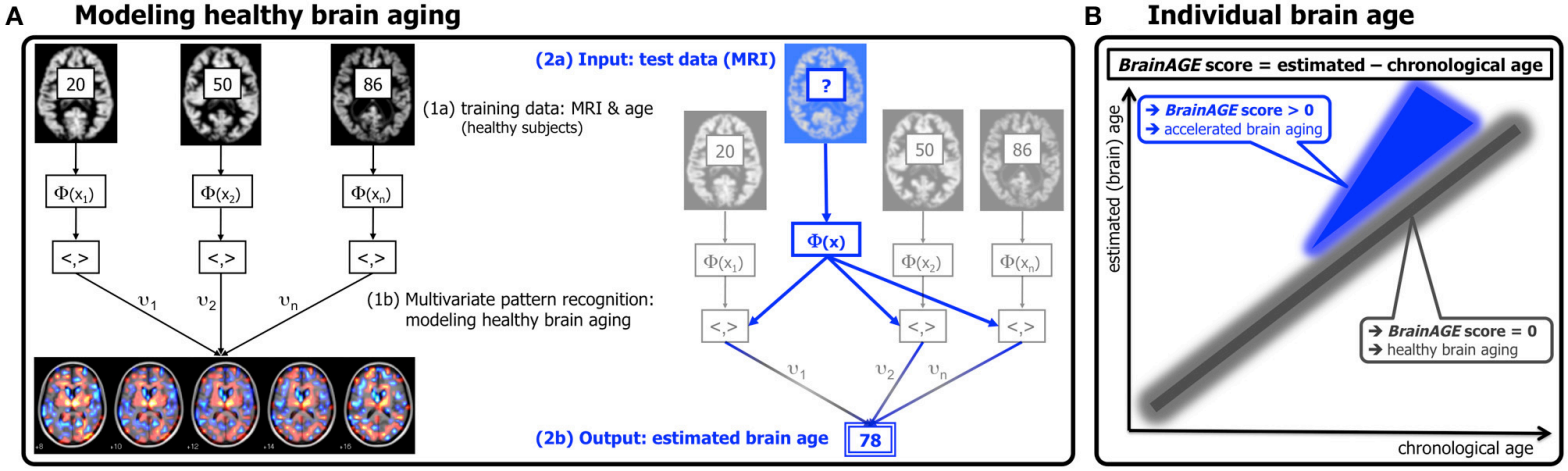
**Depiction of the *BrainAGE* concept. (A)** The model of healthy brain aging is trained with the chronological age and preprocessed structural MRI data of a training sample (left; with an illustration of the most important voxel locations that were used by the age regression model). Subsequently, the individual brain ages of previously unseen test subjects are estimated, based on their MRI data (blue; picture modified from Schölkopf and Smola, 2002). **(B)** The difference between the estimated and chronological age results in the *BrainAGE* score, positive *BrainAGE* scores indicate accelerated brain aging. (Image reproduced from Franke et al. (2012a), with permission from Hogrefe Publishing, Bern).

In general, the age regression model is trained with chronological age and preprocessed whole brain structural MRI data (as described in “Preprocessing of MRI Data and Data Reduction”) of the training sample, resulting in a complex model of healthy brain aging (Figure 1A, left panel). Put in other words, the algorithm uses those whole-brain MRI data from the training sample that represent the prototypical examples within the specified regression task (i.e., healthy brain aging). Additionally, voxel-specific weights are calculated that represent the importance of each voxel within the specified regression task (i.e., healthy brain aging). For an illustration of the most important features (i.e., the importance of voxel locations for regression with age) that were used by the RVR to model normal brain aging and more detailed information please refer Franke et al. (2010).

Subsequently, the brain age of a test subject can be estimated using the individual tissue-classified MRI data (as described in “Preprocessing of MRI Data and Data Reduction”), aggregating the complex, multidimensional aging pattern across the whole brain into one single value (Figure 1A, right panel). In other words, all the voxels of the test subject's MRI data are weighted by applying the voxel-specific weighting matrix. Then, the brain age is calculated by applying the regression pattern of healthy brain aging and aggregating all voxel-wise information across the whole brain. The difference between estimated and chronological age will reveal the individual *brain age gap estimation* (*BrainAGE*) score, with positive values indicating accelerated structural brain aging and negative values indicating decelerated structural brain aging. Consequently, the *BrainAGE* score directly quantifies the amount of acceleration or deceleration of brain aging (Figure 1B). For example, if a 70 years old individual has a *BrainAGE* score of +5 years, this means that this individual shows the typical atrophy pattern of a 75 years old individual.

Recent work has demonstrated that this method provides reliable and stable estimates (Franke et al., 2012a). Specifically, the *BrainAGE* scores calculated from two shortly delayed scans on the same MRI scanner, as well as on separate 1.5T and 3.0T scanners, produced intraclass correlation coefficients (ICC) of 0.93 and 0.90, respectively.

Within this study, the *BrainAGE* framework was applied using the preprocessed GM images (as described in the section “Preprocessing of MRI Data and Data Reduction”). For training the model as well as for predicting individual brain ages, we used “The Spider” (http://www.kyb.mpg.de/bs/people/spider/main.html), a freely available toolbox running under Matlab.

### Statistical analysis

Descriptive statistics were used to summarize all variables. Physiological and clinical chemistry parameters as markers for of individual health status were compared between the male and female sample using analysis of variance (ANOVA) for normally distributed continuous variables or Kruskal-Wallis tests for variables that were not normally distributed. Normality was tested using Shapiro-Wilk tests. Since the ADNI database includes data from about 50 different study sites across the U.S. and Canada, the *BrainAGE* scores were compared between the several sites using analysis of variance (ANOVA) to test for probable site-specific effects.

The effect of gender within the relationships between *BrainAGE* and physiological and clinical chemistry parameters were investigated by performing analysis of covariance (ANCOVA). Each specific ANCOVA included all those subjects who were measured in each specific health and lifestyle parameter, sub-grouped by gender. For each specific ANCOVA, the model fitted separate lines for the male and the female sample, thus allowing the intercept as well as the slopes to vary between both test samples.

Gender-specific effects of individual health parameters on *BrainAGE* were analyzed using linear regression models, specifically partial least squares (PLS). PLS included all subjects that were measured in all 17 physiological and clinical chemistry parameters, resulting in *n* = 107 for the male sample (mean age 75.7 ± 5.3 years) and *n* = 104 for the female sample (mean age 76.1 ± 4.8 years). However, as not all subjects were measured in all 17 physiological and clinical chemistry parameters, additional correlation analyses were performed for the most significant variables contributing to the variance in *BrainAGE* (based on the PLS variable weights) to further explore the relationships between *BrainAGE* and each of those physiological and clinical chemistry parameters. In order to control for covariates, Pearson's pairwise correlation for normally distributed variables or Spearman's for variables that are not normally distributed with adjustment for age and study site was used.

To quantify gender-specific effects of extremely low vs. extremely high levels in the most significant physiological and clinical chemistry parameters on *BrainAGE*, both test samples (i.e., male and female) were split up into quartiles for each of these clinical parameters. To illustrate the relationships between individual brain aging and extreme levels in each of these variables, the *BrainAGE* scores in the 1st quartile (lowest 25% of values) of each physiological and clinical chemistry parameter were tested against the *BrainAGE* scores in 4th quartile (highest 25% of values) of each physiological and clinical chemistry parameter, using Students' *t*-test for normally distributed parameters or Mann-Whitney test for those parameters that were not normally distributed. To control for multiple comparisons, Bonferroni-Holm correction (Holm, 1979) was applied, adjusting the *p*-value for the number of variables analyzed (i.e., 4; *p* < 0.05).

Additionally, to control for equal distribution in terms of chronological age within the 1st and 4th quartile groups as well as to explore the effects of extremely low vs. extremely high levels in the most significant physiological and clinical chemistry parameters on cognitive (i.e., Alzheimer's Disease Assessment Scale ADAS; Mohs and Cohen, 1988; Mohs, 1996) and disease severity scores (i.e., MMSE, CDR), Students' *t*-test for normally distributed parameters or Mann-Whitney test for those parameters that were not normally distributed were computed. Similar, Bonferroni-Holm-adjusted *p*-values were used to determine significance (*p* < 0.05).

Furthermore, the effect of combining the most significant variables (based on the PLS variable weights) on *BrainAGE* was explored in both test samples. Thereto, groups with “healthy” as well as “risky” clinical markers were formed. The groups with “healthy” clinical markers included all subjects who had values equal to or below the medians of the most significant physiological and clinical chemistry parameters (except for vitamin B_12_, as higher values in vitamin B_12_ are associated with more sufficient vitamin B_12_ supply and therefore “better health”). The groups with “risky” clinical markers included all subjects who had values equal to or above the medians of the most significant physiological and clinical chemistry parameters (except for vitamin B_12_, as lower values in vitamin B_12_ are associated with an insufficient vitamin B_12_ supply and therefore “poorer health”). Students' *t*-test was used to test these groups with combined “healthy” vs. combined “risky” clinical health marker values.

The Shapiro-Wilk test as well as PLS was performed using JMP 9.0 (www.jmp.com). All other testing was performed using Matlab 7.11. (www.mathworks.com).

## Results

### Group characteristics

In the male as well as in the female test sample, the mean *BrainAGE* score was 0.0 years. There were no effects for scanning sites [male: *F*_(44, 62)_ = 1.1, *p* = 0.35; female: *F*_(45, 58)_ = 1.0, *p* = 0.43]. The mean values of the physiological and clinical chemistry data evaluated here are given in Table 1. In the male test sample, only BMI, DBP, and uric acid were normally distributed. In the female test sample, albumin, SBP, DBP, cholesterol, and MCV were normally distributed. Men showed significantly higher parameter levels than women in ALT, Direct Bilirubin, Total Bilirubin, Creatinine, GGT, tHcy, Triglycerides, and Uric Acid, whereas women show significantly higher levels than men in Cholesterol and B_12_ (Table 1).

### Gender-specific effects

ANCOVAs were performed in order to investigate the effects of gender on the relationships between *BrainAGE* scores and all physiological and clinical chemistry data (Table 3). Within the whole test sample, *BrainAGE* scores varied with BMI (*p* < 0.0001), DBP (*p* < 0.01), GGT (*p* < 0.001), and uric acid (*p* < 0.01). Interactions with gender were found for ALT (*p* < 0.05), AST (*p* < 0.05), and BMI (*p* < 0.05). Thus, the effects of health parameters on *BrainAGE* show gender-specific patterns. Consequently, the following analyses were performed on males and females separately.

**Table 3:** **ANCOVA results for *BrainAGE* scores and health and lifestyle variables**.

	**Model**					
	**Gender**		**Variable value**		**Gender × value**	
	***F***	***p***	***F***	***p***	***F***	***p***
Albumin (g/dl)	0.03	0.87	0.47	0.49	0.17	0.68
ALT (U/l)	0.16	0.69	0.58	0.45	3.90	**0.05**
AST (U/l)	0.01	0.91	0.04	0.84	5.86	**0.02**
Direct Bilirubin (mg/dl)	0.09	0.77	0.46	0.50	0.21	0.65
Total Bilirubin (mg/dl)	0.56	0.45	3.74	0.06	1.23	0.27
SBP (mmHg)	0.03	0.87	1.14	0.29	0.04	0.85
DBP (mmHg)	0.06	0.80	6.40	**0.01**	0.85	0.36
BMI (kg/m^2^)	0.05	0.82	18.81	**0.0001**	4.26	**0.04**
Cholesterol (mg/dl)	0.00	0.99	0.39	0.53	0.81	0.37
Creatinine (mg/dl)	0.71	0.40	1.47	0.23	0.01	0.93
GGT (U/l)	0.44	0.51	12.49	**0.001**	0.00	0.98
Glucose (mg/dl)	0.09	0.76	0.96	0.33	0.06	0.81
MCV (fL)	0.03	0.86	0.68	0.41	0.24	0.62
TSH (μIU/mL)	0.08	0.77	3.11	0.08	0.22	0.64
tHcy (μmol/l)	0.17	0.68	3.82	0.05	0.05	0.82
Triglycerides (mg/dl)	0.07	0.79	0.00	0.96	0.25	0.62
Uric Acid (mg/dl)	1.42	0.23	6.98	**0.01**	2.52	0.11
Vitamin B12 (ng/l)	0.23	0.63	1.7	0.19	2.47	0.12

*ALT, alanin-aminotransferase; AST, aspartat-aminotransferase; BMI, body mass index; DBP, diastolic blood pressure; GGT, γ-glutamyltransferase; MCV, mean erythrocyte cell volume; SBP, systolic blood pressure; TSH, thyroid stimulating hormone; tHcy, total homocysteine; bold type = significant test results*.

### Male sample

**Figure 2:**
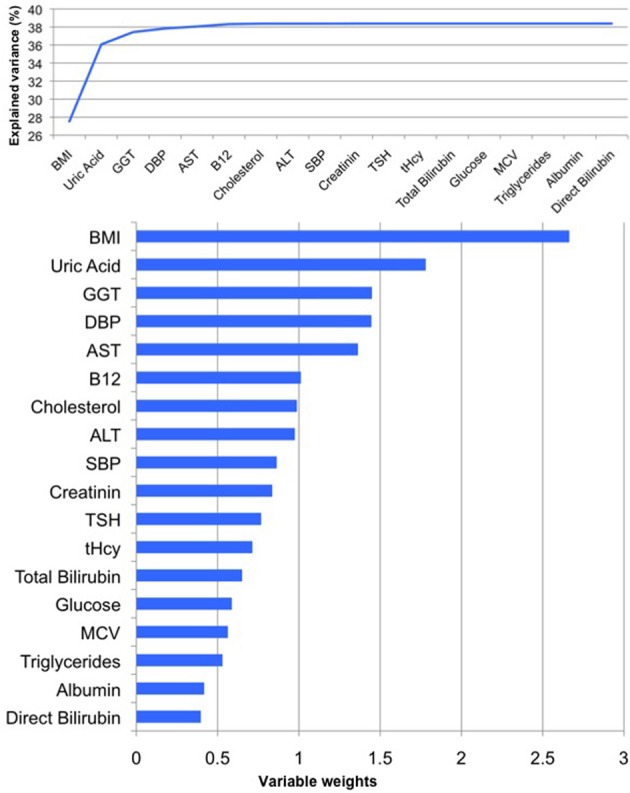
**PLS regression model of the male sample**. When modeling the relationships between *BrainAGE* and health parameters, the PLS regression model explained 39% of variance in *BrainAGE* (*p* < 0.001): BMI, uric acid, GGT, and DBP are the most significant physiological and clinical chemistry parameters as they added most to the explained variance in *BrainAGE* (top chart) and also showed the highest variable weights, i.e., the highest impact in the regression model (bottom chart).

For men, when combining all measured physiological and clinical chemistry parameters in the PLS regression model, 39% of variance within the *BrainAGE* score was attributed to the physiological and clinical chemistry parameters under consideration (*R*^2^ = 0.39, *p* < 0.001). BMI, uric acid, GGT, and DBP contributed most to the variance in *BrainAGE* (Figure 2). More specifically, higher *BrainAGE* scores were significantly correlated to higher BMI (*r* = 0.35, *p* < 0.001), increased DBP (*r* = 0.19, *p* < 0.05), increased levels of GGT (*r* = 0.20, *p* < 0.05), and increased levels of uric acid (*r* = 0.25, *p* < 0.01). This indicates a strong link between accelerated brain aging and elevated levels of these four parameters.

**Figure 3:**
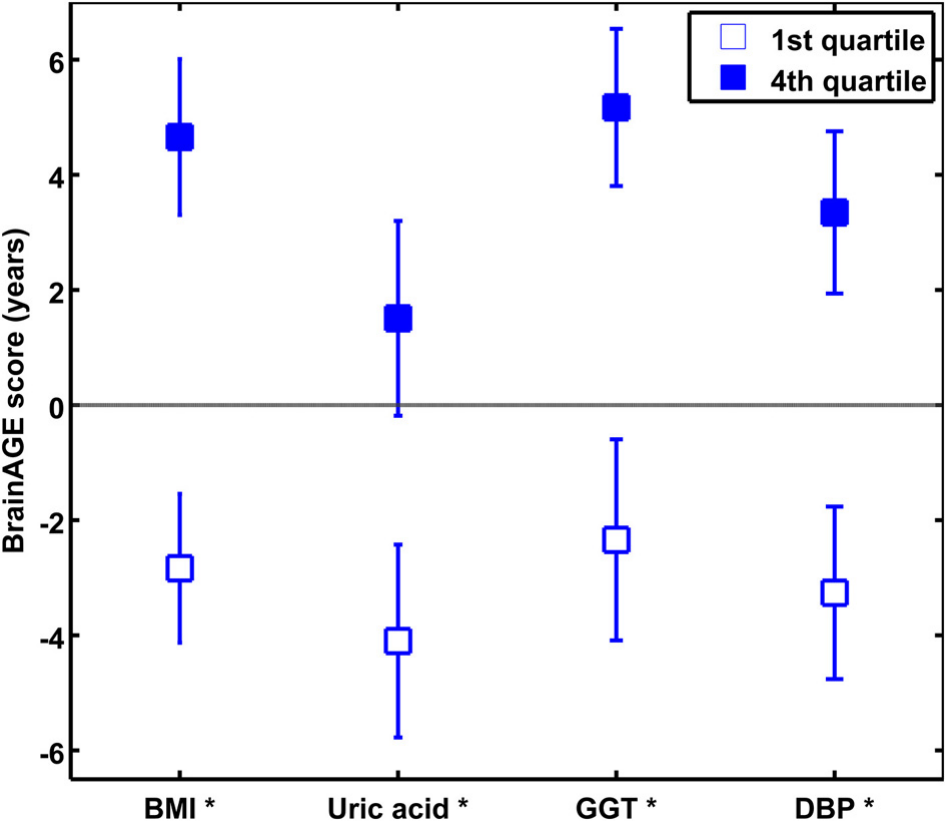
**The effects of extremely low vs. extremely high levels in clinical markers on *BrainAGE* in the male sample**. Mean *BrainAGE* scores of male subjects in the 1st (plain) and the 4th quartiles (filled squares) of the most significant physiological and clinical chemistry parameters (i.e., BMI, uric acid, GGT, and DBP). Error bars depict the standard error of the mean (SEM). [^*^p < 0.05 after Bonferroni-Holm correction].

To quantify the effects of these most significant physiological and clinical chemistry parameters on *BrainAGE*, the *BrainAGE* scores of subjects with extremely low levels (i.e., 1st quartile group) were tested against the *BrainAGE* scores of subjects with extremely high levels (i.e., 4th quartile group) in each of those four parameters (Figure 3). In all four parameters higher values were related to higher *BrainAGE* scores, thus suggesting accelerated brain aging. The absolute difference of the mean *BrainAGE* scores in the lowest vs. the highest quartile group was 7.5 years for BMI (*p* < 0.001), 6.6 years for DBP (*p* < 0.01), 7.5 years for GGT (*p* < 0.01), and 5.6 years for uric acid (*p* < 0.05). All analyses survived the Bonferroni-Holm correction. Neither chronological age, nor cognitive scores at baseline and follow-up differed between 1st vs. 4th quartile groups (Table 4).

**Table 4:** **Means (*SD*) of *BrainAGE* scores, chronological age and cognitive scores in the 1st and 4th quartile groups of the four most significant physiological and clinical chemistry parameters within the male sample**.

**MALE**	**BMI**			**DBP**			**GGT**			**Uric acid**		
	**1st quartile**	**4th quartile**	***p***	**1st quartile**	**4th quartile**	***p***	**1st quartile**	**4th quartile**	***p***	**1st quartile**	**4th quartile**	***p***
*BrainAGE* score (years)	−2.84 (6.96)	4.7 (7.29)	<0.05	−3.26 (7.03)	3.35 (7.59)	<0.05	−2.34 (9.08)	5.17 (6.83)	<0.05	−4.10 (8.03)	1.51 (8.79)	<0.05
Chronological age (years)	77.0 (6.4)	75.5 (4.1)	n.s.	78.4 (5.7)	75.3 (5.1)	n.s.	76.7 (4.8)	74.2 (4.5)	n.s.	76.4 (5.2)	74.3 (5.5)	n.s.
MMSE score (baseline)	29.1 (0.9)	29.0 (0.9)	n.s.	29.3 (0.9)	29.0 (1.0)	n.s.	28.7 (1.3)	29.1 (1.2)	n.s.	28.8 (1.1)	28.8 (1.1)	n.s.
MMSE score (12 months follow-up)	29.3 (1.0)	29.0 (1.6)	n.s.	29.3 (1.0)	28.9 (1.5)	n.s.	29.0 (1.3)	29.2 (1.1)	n.s.	29.2 (1.0)	28.8 (1.5)	n.s.
MMSE score (24 months follow-up)	29.1 (0.9)	28.8 (1.5)	n.s.	29.0 (1.1)	29.3 (1.0)	n.s.	29.2 (0.9)	28.9 (1.5)	n.s.	29.6 (0.7)	28.5 (1.6)	n.s.
MMSE score (36 months follow-up)	29.0 (1.2)	28.6 (1.4)	n.s.	29.1 (1.3)	28.8 (1.5)	n.s.	29.0 (1.2)	28.7 (1.5)	n.s.	29.3 (1.4)	28.9 (1.2)	n.s.
CDR score (baseline)	0.00 (0.00)	0.00 (0.00)	n.s.	0.00 (0.00)	0.00 (0.00)	n.s.	0.00 (0.00)	0.00 (0.00)	n.s.	0.00 (0.00)	0.00 (0.00)	n.s.
CDR score (12 months follow-up)	0.00 (0.00)	0.09 (0.20)	n.s.	0.05 (0.15)	0.04 (0.14)	n.s.	0.06 (0.17)	0.02 (0.11)	n.s.	0.03 (0.11)	0.04 (0.14)	n.s.
CDR score (24 months follow-up)	0.02 (0.10)	0.10 (0.20)	n.s.	0.05 (0.15)	0.07 (0.17)	n.s.	0.04 (0.14)	0.07 (0.18)	n.s.	0.10 (0.21)	0.06 (0.17)	n.s.
CDR score (36 months follow-up)	0.07 (0.18)	0.15 (0.28)	n.s.	0.12 (0.28)	0.08 (0.19)	n.s.	0.11 (0.21)	0.08 (0.19)	n.s.	0.00 (0.00)	0.12 (0.22)	n.s.
ADAS score (baseline)	11.3 (4.3)	10.1 (3.1)	n.s.	9.8 (4.2)	11.1 (4.0)	n.s.	9.3 (3.4)	10.6 (4.2)	n.s.	10.4 (3.1)	10.0 (3.9)	n.s.
ADAS score (12 months follow-up)	10.6 (4.2)	8.5 (4.1)	n.s.	9.4 (4.0)	10.2 (4.8)	n.s.	10.5 (5.2)	10.6 (4.2)	n.s.	10.5 (4.3)	10.6 (5.0)	n.s.
ADAS score (24 months follow-up)	10.9 (4.9)	9.6 (4.0)	n.s.	10.9 (4.8)	10.4 (4.7)	n.s.	10.5 (3.9)	10.4 (6.1)	n.s.	10.0 (4.9)	10.8 (6.2)	n.s.
ADAS score (36 months follow-up)	9.9 (4.2)	9.3 (4.6)	n.s.	9.6 (4.0)	9.0 (4.3)	n.s.	10.3 (4.4)	8.7 (5.5)	n.s.	9.2 (4.2)	10.9 (6.0)	n.s.

*p-values of t-tests after Bonferroni-Holm correction, n.s., not significant*.

*BMI, body mass index; DBP, diastolic blood pressure; GGT, γ-glutamyltransferase; ADAS, Alzheimer's Disease Assessment Scale; CDR, Clinical Dementia Rating; MMSE, Mini-Mental State Examination*.

**Figure 4:**
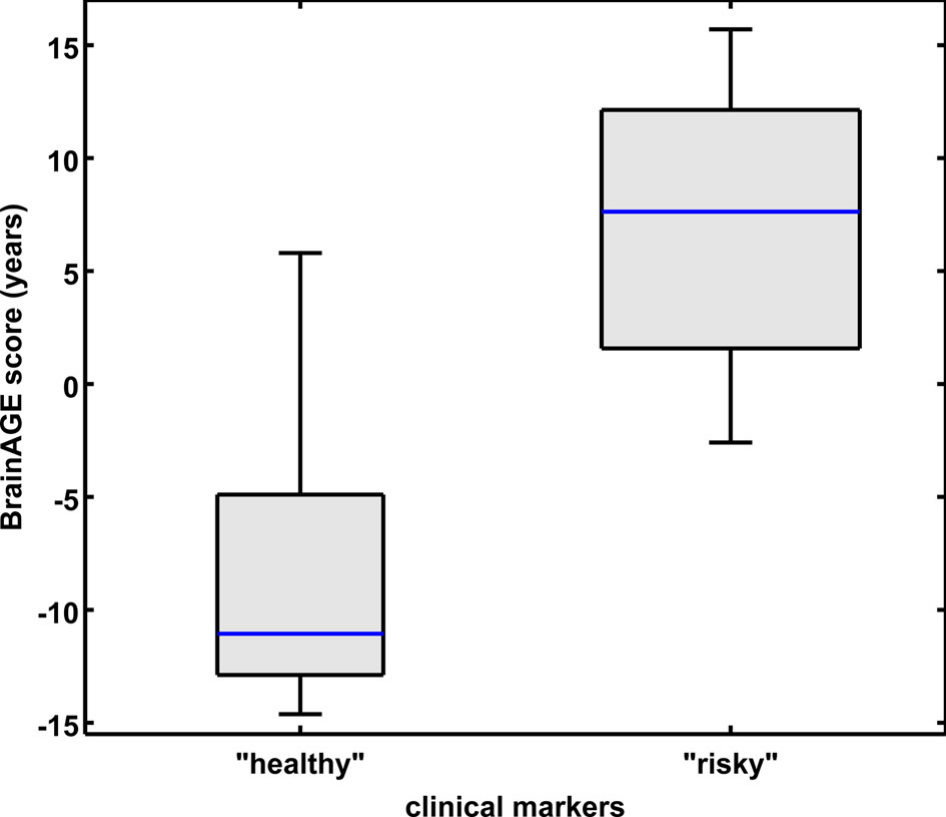
**Combined analysis of physiological and serum data parameters in the male sample**. *BrainAGE* score distributions of male subjects with “healthy” markers (i.e., values equal to or below the medians of BMI, DBP, GGT, and uric acid; *n* = 9) vs. “risky” markers (i.e., values equal to or above the medians of BMI, DBP, GGT, and uric acid; *n* = 14; *p* < 0.05). Gray boxes contain values between the 25th and 75th percentiles of the groups, including the median. Error bars indicate data within 1.5 times the interquartile range. The width of the boxes is proportional to group size.

Combining these four parameters, the effects on *BrainAGE* scores were compounded. More precisely, male subjects with values equal to or below the medians of BMI, DBP, GGT, and uric acid (“healthy” clinical markers; *n* = 9) vs. male subjects with values equal to or above the medians of BMI, DBP, GGT, and uric acid (“risky” clinical markers; *n* = 14) showed mean *BrainAGE* scores of −8.01 vs. 6.69 years, respectively (*p* = 0.015; Figure 4). However, neither chronological age, nor cognitive scores at baseline and follow-up differed between both groups (Table 5).

**Table 5:** **Means (*SD*) of *BrainAGE* scores, chronological age, and cognitive scores in male subjects with “healthy” clinical markers (i.e., values equal to or below the medians of BMI, DBP, GGT, and uric acid) vs. “risky” clinical markers (i.e., values equal to or above the medians of BMI, DBP, GGT, and uric acid)**.

**Male**	**Clinical markers**		***p***
	**“healthy”**	**“risky”**	
*BrainAGE* score (years)	−8.01 (7.11)	6.69 (6.48)	<0.05
Chronological age (years)	75.6 (5.5)	73.0 (5.2)	n.s.
MMSE score (baseline)	28.9 (0.9)	29.4 (0.7)	n.s.
MMSE score (12 months follow-up)	29.7 (0.7)	29.8 (0.6)	n.s.
MMSE score (24 months follow-up)	29.5 (0.8)	29.3 (0.9)	n.s.
MMSE score (36 months follow-up)	29.4 (0.8)	28.6 (1.7)	n.s.
CDR score (baseline)	0.00 (0.00)	0.00 (0.00)	n.s.
CDR score (12 months follow-up)	0.06 (0.17)	0.04 (0.13)	n.s.
CDR score (24 months follow-up)	0.12 (0.23)	0.00 (0.00)	n.s.
CDR score (36 months follow-up)	0.00 (0.00)	0.04 (0.14)	n.s.
ADAS score (baseline)	8.6 (4.7)	9.0 (2.9)	n.s.
ADAS score (12 months follow-up)	9.8 (4.4)	7.9 (4.1)	n.s.
ADAS score (24 months follow-up)	9.1 (4.0)	7.6 (3.3)	n.s.
ADAS score (36 months follow-up)	8.6 (4.8)	8.8 (3.4)	n.s.

*n.s., not significant*.

*ADAS, Alzheimer's Disease Assessment Scale; CDR, Clinical Dementia Rating; MMSE, Mini-Mental State Examination*.

Taken together, the results indicate a strong link between physiological and clinical health markers and structural brain aging in men, whereas no effects on cognitive scores could be found.

### Female sample

**Figure 5:**
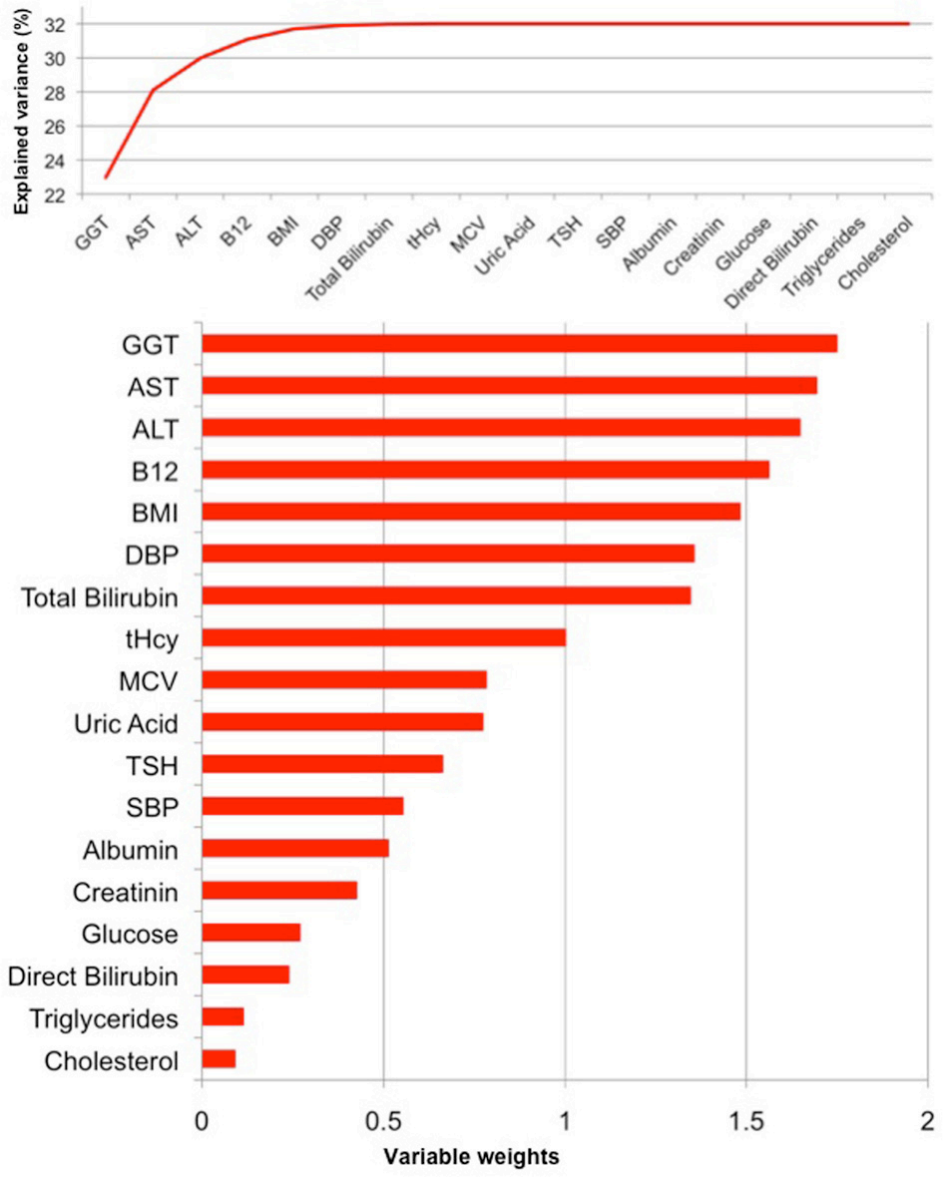
**PLS regression model of the female sample**. When modeling the relationships between *BrainAGE* and health parameters, the PLS regression model explained 32% of variance in *BrainAGE* (*p* < 0.01). GGT, AST, ALT, and vitamin B_12_ are the most significant physiological and clinical chemistry parameters as they added most to the explained variance in *BrainAGE* (top chart) and also showed the highest variable weights, i.e., the highest impact in the regression model (bottom chart).

For women, the PLS regression model combining all measured parameters was capable of explaining 32% of *BrainAGE* variance (*R*^2^ = 0.32, *p* < 0.01). As can be seen in Figure 5, GGT, AST, ALT, and vitamin B_12_ contributed most to the variance in *BrainAGE*. More specifically, higher *BrainAGE* scores were significantly related to increased levels of GGT (*r* = 0.25, *p* < 0.05), increased AST (*r* = 0.20, *p* < 0.05) and ALT levels (*r* = 0.23, *p* < 0.05), and tended to be related to decreased levels of vitamin B_12_ (*r* = −0.17, *p* = 0.08). Thus, the pattern of relationships between health and lifestyle markers and *BrainAGE* was different in the female and male samples.

**Figure 6:**
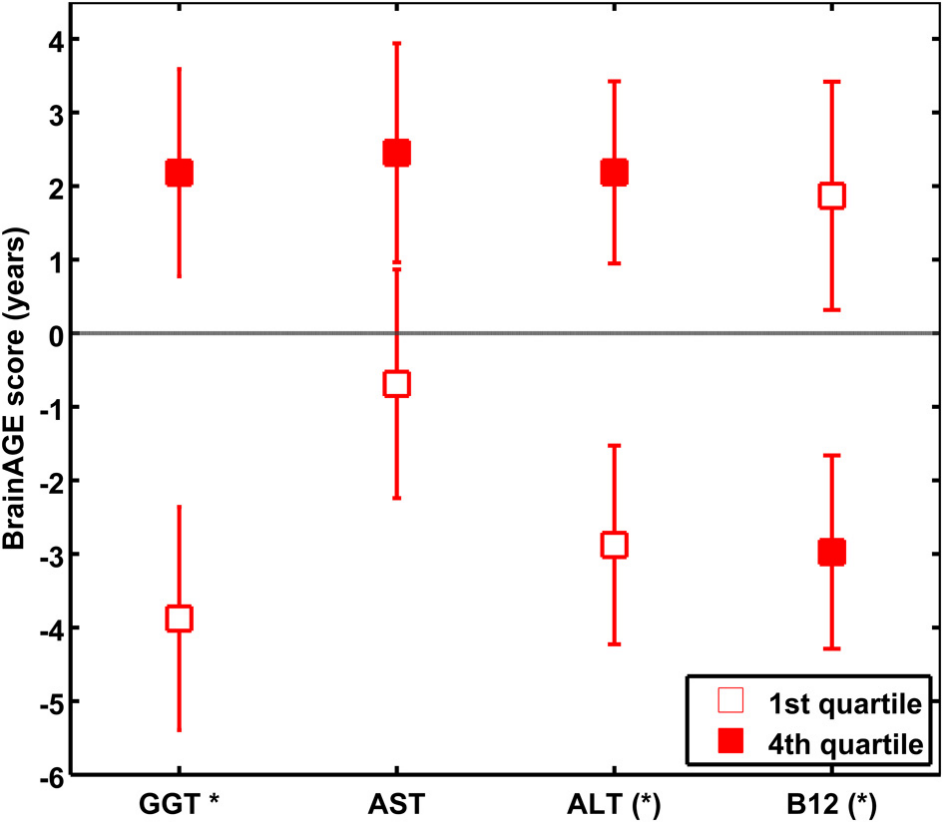
**The effects of extremely low vs. extremely high levels in clinical markers on *BrainAGE* in the female sample**. Mean *BrainAGE* scores of female subjects in the 1st (plain) and the 4th quartiles (filled squares) of the most significant physiological and clinical chemistry parameters (i.e., GGT, AST, ALT, and vitamin B_12_). Error bars depict the standard error of the mean (SEM). [(^*^) *p* < 0.05 before Bonferroni-Holm correction; ^*^p < 0.05 after Bonferroni-Holm correction].

Quantifying the effects of these four most significant physiological and clinical chemistry parameters on *BrainAGE*, the *BrainAGE* scores of subjects with extremely low levels (i.e., 1st quartile group) were tested against the *BrainAGE* scores of subjects with extremely high levels (i.e., 4th quartile group) in each of those four parameters (Figure 6). For GGT, AST, and ALT, higher parameter values were related to higher *BrainAGE* scores, thus suggesting accelerated brain aging. For vitamin B_12_, higher values were related to lower *BrainAGE* scores, thus suggesting a protective effect on brain aging. For GGT, the absolute difference of the mean *BrainAGE* scores was 6.1 years (*p* < 0.01); for AST, it resulted in 3.1 years (*p* < 0.10); for ALT in 5.1 years (*p* < 0.05); and for vitamin B_12_ in 4.8 years (*p* < 0.05). However, only GGT survived the Bonferroni-Holm correction. Neither chronological age, nor cognitive scores at baseline and follow-up differed between 1st vs. 4th quartile groups (Table 6).

**Table 6:** **Means (*SD*) of *BrainAGE* scores, chronological age and cognitive scores in the 1st and 4th quartile groups of the four most significant physiological and clinical chemistry parameters within the female sample**.

**Female**	**ALT**			**AST**			**GGT**			**B_12_**		
	**1st quartile**	**4th quartile**	***p***	**1st quartile**	**4th quartile**	***p***	**1st quartile**	**4th quartile**	***p***	**1st quartile**	**4th quartile**	***p***
*BrainAGE* score (years)	−2.88 (6.04)	2.19 (6.19)	n.s.	−0.69 (7.29)	2.45 (7.44)	n.s.	−3.88 (6.78)	2.18 (7.04)	<0.05	1.87 (8.06)	−2.97 (6.83)	n.s.
Chronological age (years)	76.8 (4.1)	76.0 (5.6)	n.s.	76.4 (4.2)	76.6 (5.9)	n.s.	77.5 (4.6)	74.5 (4.3)	n.s.	77.5 (5.0)	76.6 (4.0)	n.s.
MMSE score (baseline)	29.1 (0.8)	29.1 (1.3)	n.s.	29.0 (1.1)	29.3 (0.7)	n.s.	29.3 (0.8)	29.4 (0.8)	n.s.	29.1 (1.1)	29.4 (0.7)	n.s.
MMSE score (12 months follow-up)	28.9 (1.0)	29.2 (0.8)	n.s.	29.1 (0.9)	29.3 (0.8)	n.s.	29.3 (0.9)	29.5 (0.7)	n.s.	29.2 (1.0)	29.2 (1.2)	n.s.
MMSE score (24 months follow-up)	29.2 (0.9)	29.0 (0.9)	n.s.	29.4 (0.9)	28.9 (1.1)	n.s.	29.1 (0.8)	29.3 (0.9)	n.s.	29.1 (1.1)	29.3 (0.9)	n.s.
MMSE score (36 months follow-up)	29.5 (0.7)	28.9 (1.7)	n.s.	29.5 (0.9)	29.4 (0.9)	n.s.	29.3 (0.7)	29.3 (1.0)	n.s.	29.1 (1.5)	29.3 (0.8)	n.s.
CDR score (baseline)	0.00 (0.00)	0.00 (0.00)	n.s.	0.00 (0.00)	0.00 (0.00)	n.s.	0.00 (0.00)	0.00 (0.00)	n.s.	0.00 (0.00)	0.00 (0.00)	n.s.
CDR score (12 months follow-up)	0.00 (0.00)	0.07 (0.18)	n.s.	0.05 (0.15)	0.05 (0.15)	n.s.	0.00 (0.00)	0.05 (0.15)	n.s.	0.02 (0.10)	0.06 (0.17)	n.s.
CDR score (24 months follow-up)	0.03 (0.12)	0.03 (0.12)	n.s.	0.03 (0.11)	0.07 (0.18)	n.s.	0.00 (0.00)	0.07 (0.17)	n.s.	0.02 (0.10)	0.03 (0.11)	n.s.
CDR score (36 months follow-up)	0.11 (0.21)	0.00 (0.00)	n.s.	0.14 (0.23)	0.09 (0.27)	n.s.	0.08 (0.19)	0.09 (0.25)	n.s.	0.07 (0.18)	0.06 (0.16)	n.s.
ADAS score (baseline)	8.9 (3.7)	8.4 (3.3)	n.s.	9.8 (4.8)	9.0 (3.8)	n.s.	8.9 (4.3)	8.0 (4.6)	n.s.	9.1 (5.2)	9.3 (4.6)	n.s.
ADAS score (12 months follow-up)	7.2 (3.9)	7.2 (3.4)	n.s.	6.8 (3.3)	8.1 (3.8)	n.s.	7.2 (4.7)	7.0 (3.4)	n.s.	7.7 (3.8)	7.4 (4.3)	n.s.
ADAS score (24 months follow-up)	8.6 (5.1)	9.5 (5.6)	n.s.	8.4 (4.4)	10.5 (5.2)	n.s.	9.4 (5.1)	7.3 (4.9)	n.s.	8.1 (4.1)	8.8 (3.7)	n.s.
ADAS score (36 months follow-up)	7.5 (3.6)	7.9 (3.5)	n.s.	6.1 (3.2)	9.6 (5.1)	n.s.	7.2 (2.8)	7.6 (4.3)	n.s.	7.5 (3.6)	8.0 (3.5)	n.s.

*p-values of t-tests after Bonferroni-Holm correction; n.s., not significant*.

*ALT, alanin-aminotransferase; AST, aspartat-aminotransferase; GGT, γ-glutamyltransferase; ADAS, Alzheimer's Disease Assessment Scale; CDR, Clinical Dementia Rating; MMSE, Mini-Mental State Examination*.

**Figure 7:**
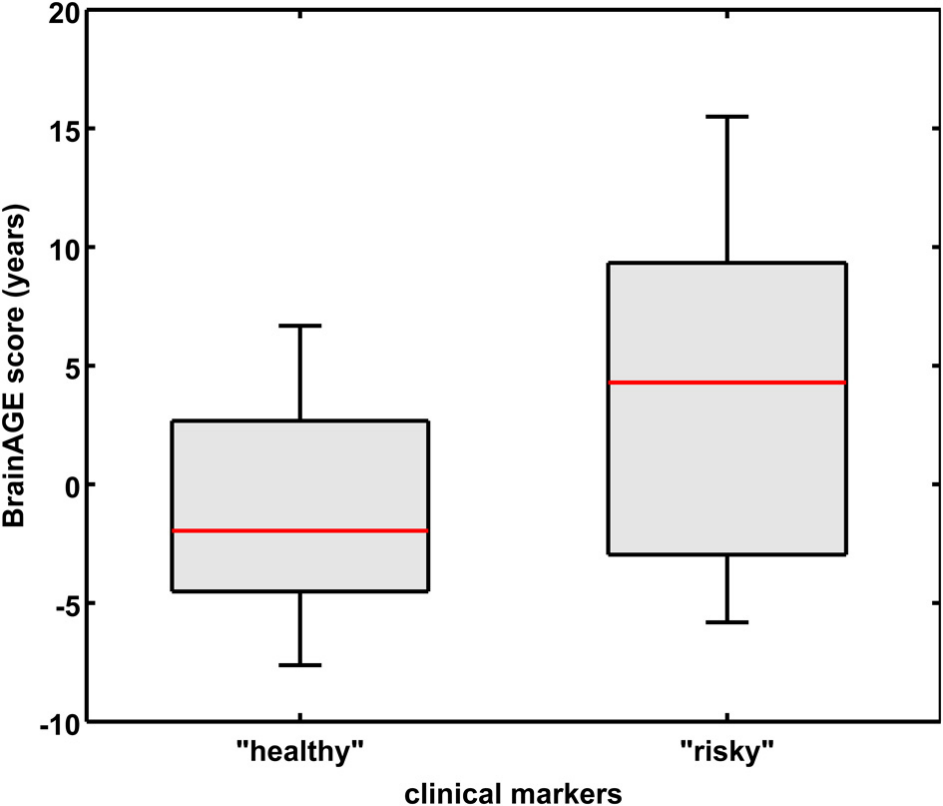
**Combined analysis of physiological and serum data parameters in the female sample**. *BrainAGE* score distributions of female subjects with “healthy” clinical markers (i.e., values equal to or below the medians of GGT, ALT, AST, and values equal to or above the median of vitamin B_12_; *n* = 14) vs. “risky” clinical markers (i.e., values equal to or above the medians of GGT, ALT, AST, and values equal to or below the median of vitamin B_12_; *n* = 13; *p* < 0.05). Gray boxes contain values between the 25th and 75th percentiles of the groups, including the median. Error bars indicate data within 1.5 times the interquartile range. The width of the boxes is proportional to group size.

As already seen in the male sample, the effects on *BrainAGE* scores were compounded when combining those four parameters. More precisely, female subjects with values equal to or below the medians of GGT, AST, ALT, as well as values equal to or above the median of vitamin B_12_ (“healthy” clinical markers; *n* = 14) vs. female subjects with values equal to or above the medians of GGT, AST, ALT, as well as values equal to or below the median of vitamin B_12_ (“risky” clinical markers; *n* = 13) showed mean *BrainAGE* scores of −0.99 vs. 3.76 years, respectively (*p* = 0.04; Figure 7). However, neither chronological age, nor cognitive scores at baseline and follow-up differed between both groups (Table 7).

**Table 7:** **Means (*SD*) of *BrainAGE* scores, chronological age, and cognitive scores in female subjects with “healthy” clinical markers (i.e., values equal to or below the medians of GGT, ALT, AST, and values equal to or above the median of vitamin B_12_) vs. “risky” clinical markers (i.e., values equal to or above the medians of GGT, ALT, AST, and values equal to or below the median of vitamin B_12_)**.

**Female**	**Clinical markers**		***p***
	**“healthy”**	**“risky”**	
*BrainAGE* score (years)	−0.99 (4.54)	3.76 (6.89)	<0.05
Chronological age (years)	76.9 (4.0)	76.6 (4.7)	n.s.
MMSE score (baseline)	29.2 (1.0)	29.4 (0.6)	n.s.
MMSE score (12 months follow-up)	29.1 (1.2)	29.3 (0.8)	n.s.
MMSE score (24 months follow-up)	29.2 (1.0)	28.7 (1.0)	n.s.
MMSE score (36 months follow-up)	29.5 (0.7)	28.8 (1.8)	n.s.
CDR score (baseline)	0.00 (0.00)	0.00 (0.00)	n.s.
CDR score (12 months follow-up)	0.04 (0.14)	0.05 (0.15)	n.s.
CDR score (24 months follow-up)	0.00 (0.00)	0.09 (0.20)	n.s.
CDR score (36 months follow-up)	0.06 (0.17)	0.15 (0.34)	n.s.
ADAS score (baseline)	7.4 (4.9)	6.6 (3.8)	n.s.
ADAS score (12 months follow-up)	7.0 (4.0)	5.6 (3.4)	n.s.
ADAS score (24 months follow-up)	8.1 (4.1)	7.1 (4.5)	n.s.
ADAS score (36 months follow-up)	6.6 (4.2)	6.9 (5.0)	n.s.

*n.s., not significant*.

*ADAS, Alzheimer's Disease Assessment Scale; CDR, Clinical Dementia Rating; MMSE, Mini-Mental State Examination*.

Similar, but to a lesser extent as seen in the men's data, these results indicate a significant link between physiological and clinical health markers and structural brain aging in women, whereas no effects on cognitive scores could be found.

## Discussion

The scope of this study was the implementation of a novel MRI-based biomarker derived from the recently presented *BrainAGE* framework to quantify the effect of several common physiological and clinical health markers on individual brain aging. Using structural MRI data, the *BrainAGE* approach aggregates the complex, multidimensional aging patterns across the whole brain to one single value (i.e., the *BrainAGE* score) and subsequently identifies pathological brain aging on an individual level. This method has been shown to accurately and reliably estimating the age of individual brains with minimal preprocessing and parameter optimization using anatomical MRI scans (Franke et al., 2010, 2012a). Additionally, higher *BrainAGE* scores were recently demonstrated to be related to measures of clinical disease severity in AD patients, as well as prospective decline in cognitive functioning (Franke et al., 2012a) and conversion to AD (Gaser et al., 2013).

In this study, the *BrainAGE* approach was applied to a new sample, which included 110 female and 118 male cognitively unimpaired elderly subjects from the ADNI database. The results provide evidence that a number of physiological and clinical health parameters have significant effects on structural brain aging, hence possibly affecting the onset of dementia. Furthermore, the effects of health measures on *BrainAGE* showed gender-specific patterns.

In cognitively unimpaired elderly men the set of physiological and clinical health markers under consideration could explain 39% of variance in *BrainAGE*. More specifically, several markers of poor health were significantly related to higher *BrainAGE* scores, suggesting advanced brain atrophy. In particular, components of the metabolic syndrome (including elevated values in BMI, DBP, and uric acid) as well as markers of impaired liver function (including elevated levels of GGT and uric acid) were significantly related to increased *BrainAGE* scores of up to 9 years. This is consistent with previous studies that associated lower total brain volume as well as an increased risk of later dementia with a higher BMI and visceral adipose tissue at mid-life (Chen et al., 2009; Fitzpatrick et al., 2009; Debette et al., 2010) and the metabolic syndrome (Enzinger et al., 2005). However, those markers were neither related to cognitive scores at baseline, nor up to three years later.

In cognitively unimpaired elderly women, 32% of variance in *BrainAGE* was explained by the set of health and lifestyle markers under consideration. In particular, markers of liver and kidney functions (including ALT, AST, and GGT) as well as vitamin B_12_ levels were related to *BrainAGE* scores. Although it still remains uncertain how vitamin B_12_ deficiency is linked to accelerated brain atrophy, cognitive decline, and dementia (McMahon et al., 2006; Aisen et al., 2008; Langan and Zawistoski, 2011), our results support recent studies, which suggested a neuroprotective role for vitamin B_12_ (Clarke et al., 1998, 2007; Czapski et al., 2012; Morris, 2012; Morris et al., 2012; Douaud et al., 2013; Hinterberger and Fischer, 2013; Kim et al., 2013). This controversy in literature regarding the effects of vitamin B_12_ on brain structure and function may be due to the heterogeneity of study samples concerning age, gender, baseline cognition, and diagnosis etc. as well as a heterogeneity of utilized analysis methods. Further, we did not find any associations between *BrainAGE* and components of the metabolic syndrome in the female sample. These results are consistent with recent studies that also found gender-specific relationships between (lifestyle-related) health markers and GM atrophy (Taki et al., 2008) or even risk for AD (Chen et al., 2009).

Even more interesting, when combining the observed gender-specific risk parameters, the effects on *BrainAGE* were profoundly compounded in the male sample. This result is in line with Luchsinger et al. (2005), reporting an increased risk of AD with increased numbers of risk factors. However, in the female sample, the compounding effect was much smaller, but still statistically significant. Additionally, the set of serum markers under consideration could explain 39% of variance in *BrainAGE* in men, opposed to 32% in women. When analyzing men and women together, only components of the PLS pattern of the male sample were significantly related to increased *BrainAGE* scores (data not shown). Thus, the present study strongly suggests distinct gender-specific patterns of brain aging associated with certain health parameters, supporting the idea of the newly founded area of gender medicine (Pinn, 2003) that the phenomenon of aging as well as the prevention, detection, treatment, and outcome of illnesses affect men and women differently, including differences in basic aspects of their normal function and their experience of the same illness. Especially for AD, it was suggested that the underlying mechanisms may be different in men and women (Grossi et al., 2005). Therefore, in the design of future studies, it should be imperative that there be enough women and men for appropriate gender-specific analyses (Azad et al., 2007).

Because this study was cross-sectional, it remains unclear whether certain health and lifestyle factors are cause or consequence of the associations found. Nevertheless, it strongly supports previously published results of personal lifestyle and overall health being related to brain health, and extends this evidence by providing a small subset of serum markers that could explain nearly 40 percent of changes in *BrainAGE*.

Even more important, this study is the first that quantified the effects of several health markers on individual brain aging in terms of years. Relying on the assumption of AD being preceded by an acceleration in brain atrophy that resembles advanced aging (e.g., Driscoll et al., 2009; Cao et al., 2010; Spulber et al., 2010; Dukart et al., 2011; Jones et al., 2011; Saetre et al., 2011), subjects with increased *BrainAGE* scores are supposed to have a greater risk for conversion to AD. However, the acceleration of spatiotemporal brain atrophy might only be seen in subjects in a preclinical stage, while in AD patients additional disease-specific pathological changes are occurring. As recently shown by Dukart et al. (2013a,b), the magnitude of GM atrophy in healthy aging was comparable to the GM atrophy associated with increasing AD symptom severity within the age range of 50–80 years. Additionally, AD symptom severity was associated with age- and symptom severity-related add-ons in GM atrophy to normal age-related atrophy. Thus, and as already shown in MCI and AD subjects (Franke et al., 2012a; Gaser et al., 2013), apparently cognitively unimpaired subjects showing accelerated brain aging may have a greater risk for prospective worsening of cognitive functions and conversion to AD. However, cognitive reserve, genetic status, education level, socioeconomic status or lifestyle may protect subjects from pathological brain aging or accelerated cognitive decline despite high *BrainAGE* scores (Snowdon, 2003; Fotenos et al., 2008; Chen et al., 2009; Querbes et al., 2009; Mangialasche et al., 2013). Future work will disentangle age- and disease-specific influences on the estimation of *BrainAGE* score to subsequently account for those influences.

Furthermore and similarly to other studies using the ADNI data and adopting its diagnostic criteria, a proportion of our sample of apparently cognitively unimpaired subjects might have a preclinical AD pathology. In our samples, seven female and 8 male subjects showed baseline MMSE scores lower than 28, thus might have a preclinical AD pathology. However, excluding those subjects from the analyses did not change the results substantially (data not shown), i.e., the patterns of gender-specific risk parameters and its relations to *BrainAGE* remained the same. Further research is therefore needed to extend our results and explore the longitudinal relationships between individual brain aging and miscellaneous factors (e.g., lifestyle, cognitive reserve, genetic status) in a larger population-based sample. Furthermore, the relationship between the duration of exposure to risk factors and accelerated brain aging, and whether reversal of modifiable factors might decelerate the progression of brain aging, should be explored.

Although applying the *BrainAGE* method results in single global estimation scores of the individual “brain age”, it accounts for the multidimensional aging pattern across all voxels in the brain. With correlations between chronological age and estimated brain age of *r* = 0.92 in healthy adults, aged 20–86 years (Franke et al., 2010), and *r* = 0.93 in healthy children and adolescents, aged 5–18 years (Franke et al., 2012b), the *BrainAGE* framework has proven to be a straightforward method to accurately and reliably estimate brain age with minimal preprocessing and parameter optimization. Most remarkably, although brain maturation in childhood as well as brain aging in late life comprise very complex, multidimensional, and highly variable processes (Good et al., 2001; Wilke et al., 2003; Lenroot and Giedd, 2006; Lebel and Beaulieu, 2011), the confidence intervals of estimated brain age did not change as a function of age (Franke et al., 2010, 2012b), underlining the potential of the approach to correctly capture the multidimensional characteristics of the different maturational and aging processes occurring in childhood and old age, respectively. Additionally, the *BrainAGE* method proved its ability to provide very stable and reliable estimates of brain aging—even across different scanners (Franke et al., 2012a). However, the *BrainAGE* framework only provides information on the magnitude of the deviation in “brain aging” from the normal aging process, but not which regions are affected and to what extent. Future work will focus on local quantitative assessment of all regional alterations in a single patient's brain to reveal information about the areas that cause the difference between estimated “brain age” and chronological age.

In summary, accelerated brain aging in cognitively unimpaired elderly subjects is related to several physiological and clinical markers of poor health, whereas a protective effect on brain aging is observed for markers of good health. Since accelerated brain atrophy was shown to precede cognitive impairment in MCI and AD (Frisoni et al., 2010; Jack et al., 2010), this study suggests that a good health, including a normal weight, appropriate liver and kidney functions, and sufficient supply of vitamin B_12_ (Steele et al., 2007; Solfrizzi et al., 2008), can prevent or at least slow down acceleration in brain aging and certain disease processes. However, gender-specific mechanisms should be taken care of in future studies.

As *BrainAGE* scores are calculated from a single T1-weighted MRI per subject, using processing techniques that can be fully automated with multi-center data, this approach may be easily implemented into clinical practice in order to encourage the identification of subtle, yet clinically-significant, changes in brain structure. With regards to health and lifestyle markers, the implications of this study may lead to a clinical tool that identifies people at risk of faster degradation of brain structure and function and potential risk for dementias, thus contributing to an early diagnosis of neurodegenerative diseases and facilitating early treatment or preventative interventions.

### Conflict of interest statement

The authors declare that the research was conducted in the absence of any commercial or financial relationships that could be construed as a potential conflict of interest.
