# Supplementary material for: Gender-specific impact of personal health parameters on individual brain aging in cognitively unimpaired elderly subjects
Source: Front Aging Neurosci. 2014 May 23;6:94. doi: 10.3389/fnagi.2014.00094 (PMC4033192; doi:10.3389/fnagi.2014.00094)
Supplement: Supplementary file 1 [file DataSheet1.DOCX]

**Supplement**

**Table S1:** Subject IDs from the ADNI database of the test samples used in this study

| **Male** | | | **Female** | | |
| --- | --- | --- | --- | --- | --- |
| 2  5  15  16  22  23  35  40  43  47  48  55  58  66  67  69  70  72  74  81  89  90  95  96  113  118  120  123  125  127  130  156  173  230  232  259  262  283  295  298  303  312  315  327 | 337  360  369  403  405  419  420  425  436  459  472  484  493  498  500  502  516  519  522  534  538  545  553  555  558  559  576  602  610  618  647  677  680  686  711  717  726  731  734  751  761  768  778  779 | 810  813  842  843  845  863  896  923  951  963  967  969  972  984  985  1009  1013  1035  1086  1094  1191  1194  1195  1200  1202  1203  1206  1241  1267  1288 | 8  14  19  21  31  56  59  61  68  86  97  106  159  166  171  172  177  184  186  196  210  223  229  245  257  260  272  301  304  311  319  352  359  363  382  386  413  416  433  441  454  467  479  488 | 489  506  520  525  526  533  548  575  578  601  605  622  637  640  643  648  657  672  681  684  685  692  741  767  818  824  862  866  876  883  886  898  899  907  920  926  931  934  981  1002  1014  1016  1021  1023 | 1063  1098  1099  1123  1133  1169  1190  1197  1212  1222  1232  1242  1249  1250  1251  1256  1261  1276  1280  1286  1301  1306 |
